# NMR spectra of oligosaccharides at ultra-high field (900 MHz) have better resolution than expected due to favourable molecular tumbling

**DOI:** 10.1016/j.carres.2006.05.017

**Published:** 2006-09-04

**Authors:** Charles D. Blundell, Michelle A.C. Reed, Michael Overduin, Andrew Almond

**Affiliations:** a Faculty of Life Sciences, University of Manchester, Manchester Interdisciplinary Biocentre, Princess Street, Manchester M1 7ND, UK; b The Henry Wellcome Building for Biomolecular NMR Spectroscopy, CR-UK Institute for Cancer Studies, University of Birmingham, Vincent Drive, Edgbaston, Birmingham B15 2TT, UK

**Keywords:** DQF-COSY, double quantum-filtered correlation spectroscopy, DSS, dimethyl-2-silapentane-5-sulfonate, FID, free induction decay, FT, Fourier transformation, GlcA, d-glucuronic acid, GlcNAc, *N*-acetyl-d-glucosamine, HA, hyaluronan, HSQC, heteronuclear single quantum correlation, NOE, nuclear Overhauser enhancement, RDC, residual dipolar coupling, ROE, rotating frame NOE, TOCSY, total correlation spectroscopy, SH2, Src homology 2, TROSY, transverse relaxation-optimized spectroscopy, Hyaluronan, Strong coupling, Resonance overlap, Spectral resolution, *T*_2_ relaxation

## Abstract

Nuclear magnetic resonance (NMR) remains the most promising technique for acquiring atomic-resolution information in complex carbohydrates. Significant obstacles to the acquisition of such data are the poor chemical-shift dispersion and artifacts resultant from their degenerate chemical structures. The recent development of ultra-high-field NMR (at 900 MHz and beyond) gives new potential to overcome these problems, as we demonstrate on a hexasaccharide of the highly repetitive glycosaminoglycan hyaluronan. At 900 MHz, the expected increase in spectral dispersion due to higher resonance frequencies and reduction in strong coupling-associated distortions are observed. In addition, the fortuitous molecular tumbling rate of oligosaccharides results in longer *T*_2_-values that further significantly enhances resolution, an effect not available to proteins. Combined, the resolution enhancement can be as much as twofold relative to 600 MHz, allowing all ^1^H-resonances in the hexasaccharide to be unambiguously assigned using standard natural-abundance experiments. The use of ultra-high-field spectrometers is clearly advantageous and promises a new and exciting era in carbohydrate structural biology.

## Graphical abstract

**Figure:**
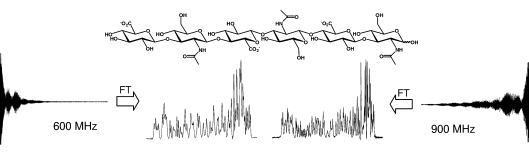

In contrast to most globular proteins, the three-dimensional structure of complex carbohydrate molecules in solution cannot be approximated to a single well-defined conformation. Rather, carbohydrates are believed to adopt a range of dynamically interchanging conformations in solution. It is therefore necessary to measure the range and frequency of all molecular motions if a fair depiction of the solution conformations of a carbohydrate is to be achieved.^1^ Crucially, unless carbohydrate structures can be described in this way, our understanding of the biology that arises from their structural properties will be limited. Nuclear magnetic resonance (NMR) provides the best potential to acquire such dynamical structural information, if only spectral resolution can be obtained, but other techniques such as molecular dynamics simulations and hydrodynamics also play a vital role.^2,3^

Also unlike globular proteins, carbohydrate molecules generally adopt open, extended structures in solution. A particular consequence of this is that few (NMR-active) ^1^H nuclei are close enough to each other to experience nuclear Overhauser enhancements (NOEs), the main experimental observable used to define protein structures. This is particularly problematic because considerably more experimental restraints are needed to define dynamic structures than static ones. As a result, other observables such as rotating frame NOEs (ROEs), residual dipolar couplings (RDCs) and conformational-dependent scalar couplings are sought.^2,4–6^ However, unless sufficient spectral resolution is achieved, these restraints can either not be obtained or are measured inaccurately.

Even at high field (up to 600 MHz proton frequency) the lack of spectral resolution that arises from the repetitive chemical structure of complex carbohydrates is the main obstacle to their study by NMR.^7^ The most distinct ^1^H resonances come from the anomeric protons (i.e., those attached to the linkage carbon), and resolution is usually achieved by correlating other nuclei to them.^7,8^ However, for oligosaccharides derived from polysaccharides, which comprise repeated units, even this resolution is lost; these are therefore excellent test-cases for the development of techniques to achieve spectral resolution. One of the most challenging polysaccharides to study is hyaluronan (HA), comprising a repeated disaccharide of *N*-acetyl-d-glucosamine (GlcNAc) and d-glucuronic acid (GlcA) (Fig. 1). Unlike other glycosaminoglycans, HA contains exclusively β-linkages and does not have any resolution-promoting variable sulfation or epimerization. As a result, complete ^1^H assignment of HA oligosaccharides has only been achieved before for tetrasaccharides (at 600 MHz) using chemical modification (and not without disagreement).^9,10^ However, using simple experiments at 900 MHz (COSY, TOCSY, ^13^C-HSQC) and without recourse to chemical modification, isotope-enrichment or complicated pulse-sequences (see, e.g., Armstrong et al.,^11^), we have assigned all ^1^H resonances within HA hexasaccharides (Fig. 1), which was not possible before, even at 750 MHz.^12^ The ease with which this was possible has prompted us to draw attention to the specific benefits of ultra-high-field NMR to the wider carbohydrate community. To the best of our knowledge, there is currently only one previous report of NMR data recorded on oligosaccharides at such high field.^13^

The first and most obvious advantage of NMR at higher magnetic field is the greater intrinsic separation of resonances. While the chemical shift is invariant to changes in spectrometer magnetic field-strength (*B*_0_), the resonant frequencies of nuclei, and hence their observed differences in hertz, scale linearly with *B*_0_. Moreover, homonuclear scalar couplings remain constant with *B*_0_ and therefore complex multiplets overlap less with neighbouring resonances at higher field-strengths; this also allows their fine-structure to be interpreted more easily. In addition, the greater energy of nuclear spin-state transitions associated with the higher *B*_0_ results in a greater difference in the net magnetization available for manipulation (according to the Boltzmann distribution), significantly improving the signal-to-noise ratio achievable. These gains in spectral resolution, solely due to higher field-strengths, significantly clarify carbohydrate NMR spectra.

Perhaps less obvious to the uninitiated is the effect of higher field-strength on resonance linewidths. The NMR signal induced in the receiver coil (i.e., the free induction decay, FID) arises from magnetization in the transverse plane and relaxes via spin–spin relaxation mechanisms, which are characterized by an exponential decay with time constant *T*_2_. The transformed FID gives Lorentzian lineshapes, with the linewidth at half-height being inversely proportional to *T*_2_ (provided that other things, such as field homogeneity, are equal). The major determinants of *T*_2_ are the overall rotational correlation time, internal motion and *B*_0_. The overall rotational correlation time (*τ*_m_; colloquially referred to as the ‘tumbling time’) represents the time it takes for a molecule to reorient by one radian and is inversely related to *T*_2_. Large molecules, such as globular proteins, tumble slowly (on the order of 10 ns), giving rise to fast *T*_2_ relaxation and hence resonances with broad line-widths. Small and flexible molecules, such as oligosaccharides, tumble much more quickly (on the order of 0.1–1 ns), giving rise to slower *T*_2_ relaxation and hence much sharper resonances. Moreover, the dependence of *T*_2_ with *B*_0_ for molecules with fast *τ*_m_ is such that at higher field-strengths *T*_2_ relaxation is actually more favourable, giving yet sharper lines. In contrast, the higher overall correlation times associated with proteins provide negligible gain in linewidth due to spin–spin relaxation alone. Re-sharpening of protein linewidths at ultra-high field therefore has to be achieved by offsetting dipole–dipole and chemical shift anisotropy relaxations around ^15^N nuclei in isotopically-labelled proteins using TROSY-NMR.^14,15^

To illustrate the advantage of slower *T*_2_ relaxation, the FIDs for 1D spectra of a sample of HA hexasaccharide collected under comparable conditions at 600 and 900 MHz are compared in Figure 1; the exponential rate of decay of the FID is clearly much greater at 600 MHz. The corresponding frequency domain of the most crowded region (i.e., 3.7–4.0 ppm) is shown, highlighting the fact that the decreased linewidth considerably helps spectral resolution. For example, the linewidth at half-maximum of each component of the doublet of the GlcA H-1^II^ proton in HA hexasaccharide (*δ* 4.506 ppm) decreases from 1.7 to 1.4 Hz upon increasing *B*_0_ from 600 to 900 MHz (see Fig. 2). Because chemical shift differences that need to be resolved in repetitive oligosaccharides derived from polysaccharides like HA are typically ∼0.002 ppm (equivalent to 1.2 and 1.8 Hz at 600 and 900 MHz, respectively), reduction of linewidths in this manner is an important advantage of moving to higher fields. A further benefit of narrower resonance linewidths that should not be overlooked is the concomitant gain in signal-to-noise (∼20-fold better in this case).

Spin–spin dipolar relaxation rates can be calculated using standard equations that depend on a theory of molecular tumbling and internal motion to describe the spectral density function;^16^ typically the Lipari–Szabo model-free approach^17^ is used for this purpose. Figure 2 shows the relationship between *T*_2_ relaxation time and overall correlation time at high and ultra-high field-strengths. It can be seen that at short overall correlation times (less than 1 ns) there is a significant advantage in linewidth to be gained from increases in *T*_2_ longevity, which corresponds to overall correlation times associated with oligosaccharides and peptides. However, for overall correlation times greater than 5 ns (i.e., those typically associated with proteins) there is little to be gained. This extra resolution gain due to effects of *T*_2_ variability between 600 MHz and 900 MHz has a maximum contribution of a factor of ∼1.25 at a *τ*_m_ value of 0.25 ns (corresponding to di- and tetrasaccharides).

Theoretically, resolution can be described by consideration of the spectral resolution of two peaks (*R*_*ν*_), which is defined as their separation divided by their linewidths (both in hertz). The separation of peaks is proportional to *B*_0_ and hence the nuclear spin Larmor frequency (*ν*). Because a processed resonance is the Fourier transform of an exponential FID decay, the lineshape is by default a Lorentzian with a full-width at half-maximum of (π*T*_2_)^−1^ (i.e., inversely proportional to *T*_2_). Therefore, the resolution (*R*_*ν*_) can now be seen (Eq. 1) to depend on a product of the resonant frequency (*ν*) and *T*_2_.

(1) $$R_{\nu }\propto T_{2}B_{0}\propto T_{2}\nu$$

The resolution enhancement (i.e., *R*_900_/*R*_600_) predicted due to moving to higher magnetic field-strengths is shown in Figure 2 (bottom panel) for two molecules, a small, flexible oligosaccharide with *τ*_m_ = 0.63 ns (as measured previously for a HA hexasaccharide at ring III^1^) and a typical protein domain with an overall correlation time (*τ*_m_) of 9.2 ns (C-terminal SH2 domain of phospholipase Cγ1^16^). While the larger and less flexible protein domain only gains resolution from the relative spectrometer *B*_0_ frequencies (i.e., *R*_900_/*R*_600_ = 1.5), the small and flexible sugar molecule is expected to have a greater resolution enhancement (*R*_900_/*R*_600_ ≈ 1.7), that is, more than would be expected based on the relative spectrometer frequencies. The experimentally observed value for the GlcA H-1^II^ proton (detailed above, see Fig. 2) compares favourably with this expectation, having an *R*_900_/*R*_600_ value of 1.8 [(1.7 Hz/1.4 Hz) × (900 MHz/600 MHz)]. A greater resolution enhancement is achieved experimentally due to the limitations of the Lipari–Szabo model-free approach, which approximates all motions to be either internal or overall, that is, discounting any intermediate motions that undoubtedly occur. This can be compared to the theoretical maximum resolution enhancement of ∼1.9 at an overall correlation time of 0.25 ns due to both increased magnetic field-strength and slower *T*_2_ relaxation (i.e., twice the resolution for only a 50% increase in field-strength). Large oligosaccharides or polysaccharides (with overall correlation times greater than or equal to proteins) will not derive any resolution enhancement from their overall correlation times. However, internal dynamics on sub-nanosecond timescales (i.e., segmental motions) may still result in slower *T*_2_ relaxation and hence sharper resonance linewidths.

Another frequently encountered feature of carbohydrate NMR is strong coupling, which generates distortions to resonance lineshapes not predicted at first order. Linear combinations of pure states (such as ∣αβ> and ∣βα>) occur in the full quantum description of NMR, but when the so-called weak-coupling limit is satisfied, these terms become negligible. The condition for weak coupling is shown in Eq. 2, that is, when the difference in frequency (chemical shift × *B*_0_ frequency) between two nuclei (Δ*ν*_IS_) is considerably greater than the scalar coupling (*J*_IS_) between them.

(2) $$\Delta \nu_{\mathrm{IS}}=|\nu_{I}-\nu_{S}|\gg J_{\mathrm{IS}}$$

The weak-coupling limit is usually valid for protein molecules, which generally have well-dispersed proton resonances with relatively few large scalar couplings (>10 Hz) between them. However, when the weak coupling limit is not satisfied, the nuclei are said to be strongly coupled, and distorted lineshapes and artifacts due to non-pure quantum states arise. Such features plague carbohydrate NMR spectra because sugar rings contain protons that are clustered within a narrow spectral region (i.e., 3–4 ppm) and in glucose, galactose and mannose derivatives there are many *trans* vicinal protons, which have large scalar couplings between them (∼10 Hz, equivalent to 0.017 and 0.011 ppm at 600 and 900 MHz, respectively). These distortions not only complicate interpretation of spectra but, more significantly, they prevent accurate measurement of peak volumes and frequencies, which is crucial if NOEs, ROEs and RDCs are to be used as structural restraints. At higher field-strengths, however, the differences in hertz between scalar-coupled protons increases while the coupling remains constant, that is, diminishing the effects of strong-coupling on spectral quality. As an example, the multiplet structure of GlcA H-2^VI^ (from 1D spectra) is shown at both 600 and 900 MHz in Figure 3. While this proton shows the expected quartet from the two vicinal ^3^*J*_HH_ couplings (i.e., ^3^*J*_1,2_, ^3^*J*_2,3_), strong coupling between GlcA H-3^VI^ and GlcA H-4^VI^ produces virtual coupling ‘wings’^18^ at GlcA H-2^VI^. The intensity of these wings relative to the first-order resonances (i.e., those from ^3^*J*_1,2_ and ^3^*J*_2,3_) is considerably less at 900 MHz and the greater resolution achieved (through the combination of increased *B*_0_ field and reduced linewidth) allows this portion of the spectrum to be deciphered considerably more easily.

The combined effect of all these advantages of carbohydrate NMR at high field are illustrated in a comparison of 2D DQF-COSY spectra of the crowded region of HA hexasaccharide, recorded on a single sample in an identical manner at both 600 and 900 MHz (Fig. 4). The most obvious contribution to the gain in resolution is the decreased spectral width of each resonance, allowing the COSY correlations to be distinguished more clearly. Two of the most overlapped portions of the spectrum are highlighted (A–D, E–G) to draw attention to the gain in resolution achieved. It is clear from region A–D that the sharper lines (arising from the longer *T*_2_ relaxation time) are particularly helpful in resolving extremely overlapped peaks where the differences in chemical shift are comparable to the multiplet component linewidths. Peaks E, F and G arise from the GlcNAc geminal hydroxymethyl protons on rings V, III and I (β-anomer), respectively, which cannot be distinguished at 600 MHz. In contrast, at 900 MHz, even this portion of the spectrum is interpretable, which is remarkable considering the differences in chemical shifts between these resonances are only ∼0.010 ppm (see Fig. 4 legend). Strong coupling artifacts are also reduced at 900 MHz, for example the H-2^VI^ (*δ* 3.318 ppm) to H-5^VI^ (3.722) COSY cross-peak^9^ (Fig. 4, H) significantly decreases in intensity relative to the H-2^VI^ diagonal peak (by 33%). In combination with standard TOCSY and ^13^C-HSQC spectra, these increases in spectral resolution have allowed us to unambiguously assign all ^1^H resonances within HA hexasaccharide (manuscript in preparation). It is therefore now possible to acquire a larger number of resolved structural restraints to produce a better description of the range and rate of change of conformations adopted by HA in solution.

The increasing number of 900 MHz magnets available globally means that their use in carbohydrate NMR can become more commonplace. Moreover, the capability to run simple and standard spectra to achieve assignment and acquisition of structural restraints, without having to resort to labour-intensive isotopic enrichment, derivatization, chemical synthesis or specifically tailored NMR pulse-sequences means that such use is a viable cost-effective solution for carbohydrate analysis. It could be argued that the large increases in effective resolution, which can be as great as twofold for complex carbohydrates, offsets some of the cost differential between traditional high-field (600 MHz) and ultra-high-field NMR (900 MHz and beyond). These new technologies promise to revolutionize the NMR of flexible carbohydrates and initiate a new era in understanding their structural biology.

## 1 Experimental

All experiments were performed at 24.6 °C on a single 5 mM HA hexasaccharide sample (>95% purity, 10% D_2_O, 0.02% NaN_3_, 0.5 mM DSS, pH 6.0), prepared as described previously.^19^ Identical NMR datasets were recorded using Varian INOVA 600 and 900 MHz spectrometers (^1^H resonance frequencies of 599.8 and 899.9 MHz) equipped with room temperature 5 mm triple-resonance pulse-field gradient probes, scaling dwell-times as appropriate. Considerable effort was undertaken to ensure that the *B*_0_ field was as homogenous as possible by shimming. 1D spectra were acquired over 16,384 complex points with dwell-times of 104.0 μs (600 MHz) and 68.9 μs (900 MHz). DQF-COSY spectra were collected with 768 complex points in *t*_1_ and dwell-times of 178.6 μs (600 MHz) and 119.0 μs (900 MHz) and, in *t*_2_, 1365 (138.7 μs, 600 MHz) or 2048 complex points (92.5 μs, 900 MHz). Spectra were processed to obtain the maximum resolution possible from each dataset using, where possible, identical combinations of linear prediction, window functions (cosine-bell and Lorentz-to-Gauss transformations, apodizing to ∼5% of the initial intensity) and extensive zero-filling.^19^

## Figures and Tables

**Figure 1:**
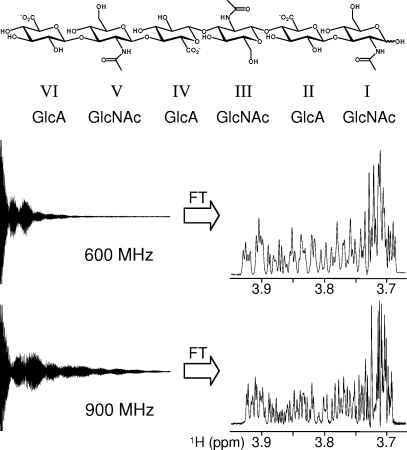
(Top) Chemical structure of a hyaluronan (HA) hexasaccharide, which comprises three repeats of a disaccharide of d-glucuronic acid (GlcA) and *N*-acetyl-d-glucosamine (GlcNAc). Ring positions are numbered from the reducing terminus. (Bottom) Comparison of free-induction decays of HA hexasaccharides at 600 and 900 MHz (720 ms shown in both cases). Upon Fourier-transformation (FT), the smaller rate of exponential decay (i.e., longer *T*_2_ relaxation time) at 900 MHz results in narrower line-widths and a concomitant increase in resolution (the most crowded region is shown, from 4.0 to 3.7 ppm).

**Figure 2:**
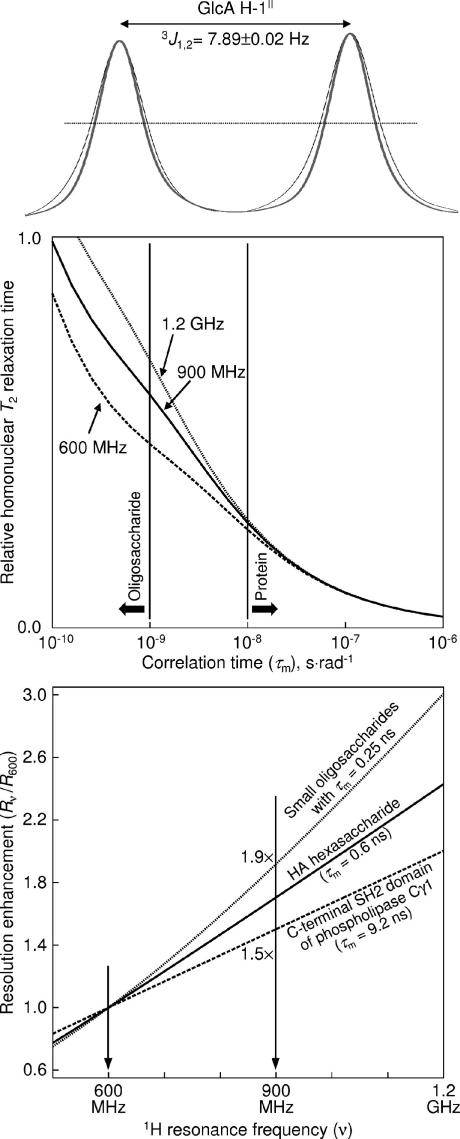
(Top) Comparison in hertz of line-widths at half-maximum (dotted line) for the GlcA H-1^II^ doublet at 600 MHz (black) and 900 MHz (grey). (Middle) Dependence of *T*_2_ relaxation rate on the overall correlation time (*τ*_m_) at high field (600 MHz) and ultra-high fields (900 MHz and 1.2 GHz). Regions typically associated with oligosaccharides and proteins are marked. (Bottom) Comparison of the resolution enhancement (*R*_*ν*_/*R*_600_) gained by increasing the *B*_0_ field-strength for small oligosaccharides (with *τ*_m_ = 0.25 ns), a HA hexasaccharide with segmental motion (mass ∼1 kDa, *τ*_m_ = 0.63 ns, *τ*_e_ = 0.071 ns and *S*^2^ = 0.57, as measured previously at ring III^1,20^) and a protein with a mass of ∼12 kDa (*τ*_m_ = 9.2 ns, *τ*_e_ = 0.02 ns and *S*^2^ = 0.94, as measured for Ala33 in the C-terminal SH2 domain of phospholipase Cγ1^16^).

**Figure 3:**
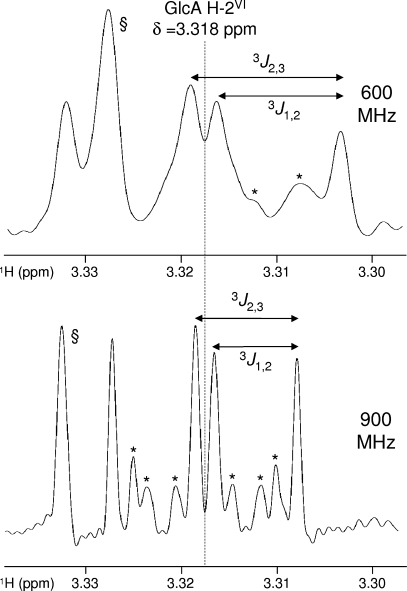
Strong coupling between GlcA H-3^VI^ and GlcA H-4^VI^ results in virtual coupling wings^18^ (∗) to the GlcA H-2^VI^ quartet. The intensity of these wings relative to the first-order resonances (i.e., those from ^3^*J*_1,2_ and ^3^*J*_2,3_) is considerably less at 900 MHz. The resonance indicated by § is part of the GlcA H-2^IV^ multiplet.

**Figure 4:**
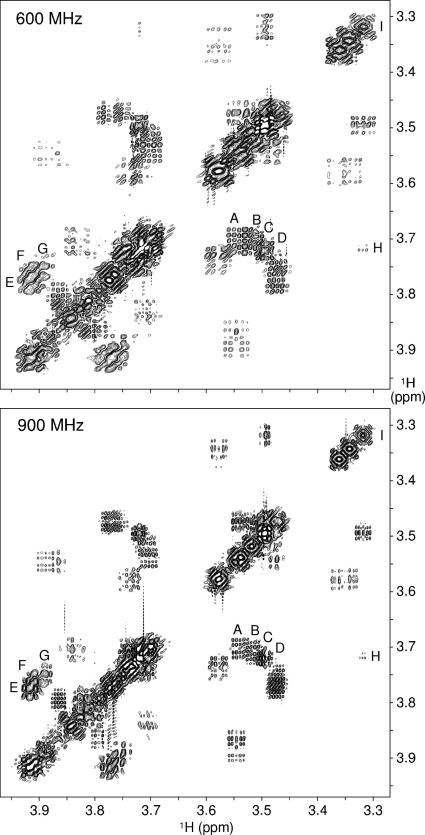
Comparison of DQF-COSY spectra recorded in an identical manner on a sample of HA hexasaccharide at 600 and 900 MHz. Several cross-peaks that are considerably easier to assign at 900 MHz are indicated (A–G), as well as an artifact caused by strong coupling (H). A—GlcNAc, H-4^V^, 3^V^ (*δ* 3.539, 3.705 ppm); B—GlcNAc, H-4^III^, 3^III^ (3.519, 3.707); C—GlcNAc, H-4^I^, H-3^I^ (3.513, 3.714) (β anomer); D—GlcA, H-4^VI^, H-5^VI^ (3.498, 3.722); E—GlcNAc, H-6b^V^, H-6a^V^ (3.918, 3.776); F—GlcNAc, H-6b^III^, H-6a^III^ (3.910, 3.764); G—GlcNAc, H-6b^I^, H-6a^I^ (3.891, 3.749) (β anomer); H—GlcA, H-2^VI^, H-5^VI^ (3.318, 3.722); I—GlcA, H-2^VI^ diagonal peak (3.318).
